# Why do nurses leave their jobs? Understanding person-related hostility in the healthcare sector of Pakistan

**DOI:** 10.1371/journal.pone.0298581

**Published:** 2024-06-03

**Authors:** Nadia Noor, Saqib Rehman, Yasmeen Ahmed, Sohail Rizwan, Muhammad Sarmad

**Affiliations:** 1 Department of Management Sciences, Lahore College for Women University, Lahore, Pakistan; 2 Department of Architecture, Lahore College for Women University, Lahore, Pakistan; 3 Department of Commerce, Fatima Jinnah Women University, Rawalpindi, Pakistan; 4 Riphah School of Leadership, Riphah International University, Islamabad, Pakistan; National University of Sciences and Technology, PAKISTAN

## Abstract

Nursing is considered indigent and oppressed because of uneven organizational hierarchies and unsatisfactory work environments. This study aimed to highlight the critical aspects of organizational culture in the nursing profession and, in general, those propagating hostile behaviours among female nursing staff that result in dissatisfaction and intention to leave the organization. A quantitative research approach was applied and a survey research strategy was used to collect the data. Convenience sampling was applied and data were collected from female nurses who were easily accessible and willing to participate in the research. A total of 707 questionnaires were collected from 14 hospitals and the data was analyzed using SmartPLS 4. Lack of administrative support and gender discrimination positively affected person-related hostility. In contrast, person-related hostility mediated the relationship between gender discrimination and lack of administrative support with the intention to leave. Direct or indirect person-related hostility factors can severely damage organizational reputation and quality and may cause the loss of employees with specific organizational knowledge and exposure. Losing an experienced employee to a newer one cannot replace the costs incurred on hiring, training, and providing knowledge to older employees. HR managers in organizations should devise strategies and policies that allow for the timely resolution of issues of nursing staff based on fair work performance.

## 1. Introduction

Mutual and respectful communication among colleagues is essential for evaluating, managing, and ensuring healthcare quality. For the last many decades, descriptive and informal data on communication among professionals exhibit the opposite of these standards and morals [1]. In particular, horizontal hostility in the healthcare setting negatively impacts intrinsic and extrinsic satisfaction and positively correlates with burnout and poor psychological health indicators in the nursing workforce [2]. Moreover, workplace dissatisfaction and hostility have indirectly affected patient care quality and resulted in threats to healthcare standards [3] in the form of employee exit [4]. Horizontal hostility has been ingrained in the patriarchal historical background of the nursing profession, with internalized sexism generating oppression, both individually and collectively. Individuals who feel oppressed hurt their coworkers to regain power informally [5]. Nurses face oppression due to external influences of physicians, administrators, and nurse managers which make them feel a lack of empowerment and control over their professional roles [6, 7].

The problem of nurses leaving their jobs because of horizontal hostility in the healthcare sector can be viewed from different aspects. Firstly, in underdeveloped countries, women face significant professional barriers due to a lack of administrative support and gender discrimination, which often leads to deficient job opportunities [8]. Secondly, in male-dominated societies, males often resist female managers or supervisors who command them, leading to decreased support at work [9]. Thirdly, in developing nations, organizations neglected to understand and acknowledge the fact that men and women have practically equal opportunities for advancement in their careers and all fields of life, including healthcare, nursing, the media, the hospitality industry, and other non-profit work [10]. Due to this, there is a persistent dearth of female representation in senior administration and executive roles, and the recruiting system of these countries idealizes men with the perception that only men have the expertise and superior education to perform certain jobs [11]. Fourthly, gender discrimination in the workplace causes stress, and studies have shown that women who experience it are more likely to lash out at coworkers of the same gender or show person-related hostility and quit their jobs altogether [12].

According to [13] and [14] horizontal hostility is a complex and multicultural issue that has significant psychological effects on both patients and nursing recipients in the healthcare industry. [15] argued that to address the issue, it is necessary to study its occurrence in other cultural contexts with relevant antecedents and outcomes. Horizontal hostility, also known as nonphysical intergroup conflict, manifests itself in the nursing profession as both overt and hidden hostile behaviours [16, 17]. High nurse turnover, absenteeism, lower-quality patient care, and less productivity are some of the potentially disturbing outcomes [13]. To effectively manage the factors that become the reason for these harmful behaviours in the nursing profession, more study is needed [18, 19]. To ensure a safe and peaceful workplace where quality patient care is observed, it is essential to identify and eradicate horizontal hostility at all administrative levels so that others (peers) may not be affected [20]. Horizontal hostility has three dimensions including work-related hostility, person-related hostility, and physically intimidating hostility. Person-related hostility has never been explored in developing nations’ context, especially with employee-level outcomes like intention to leave. In light of these highlighted gaps, it is imperative to study the root causes of horizontal hostility at the organizational level to foster a work climate that discourages employees from leaving the organizations and provides high-quality healthcare standards.

In Pakistan, female nurses in public-sector healthcare settings experience mistreatment and hostility from their supervisors, colleagues, and patients. An unmanageable workload makes them feel stressed and overburdened [21]. Staff shortages, stereotypes regarding the nursing profession, insufficient medical equipment, poor administration, and less support result in stress, conflict, poor professional care, and compromised patient safety [21]. The study provided valuable insights for human resource professionals, enabling them to develop strategies to address employee turnover, improve administrative support, and foster positive relationships, ultimately enhancing employee satisfaction and retention. This study also offered insights for academia and organizations in Pakistan to improve workplace environments by contextualizing variables within the country’s specific dynamics. The study aims to shed light on the significant aspects of organizational culture in the nursing profession. These aspects include a lack of administrative support and gender discrimination, which turn female employees toward hostile behaviour against other females in the workplace and cause an organizational loss in the form of dissatisfied employees, whereas organizational fairness can be a solution to stop leaving employees [22].

## 2. Literature review and hypotheses development

Friere’s "Pedagogy of the Oppressed" [23] explored the aggressive behavior of oppressed minorities in developing countries, who attack their peers instead of confronting their oppressors. Since the 1970s, feminists have used the phrase "horizontal hostility" to refer to internal conflicts or divisions within the women’s movement. Horizontal hostility characterizes power as female dominance. They exhibit negative behaviors and a display of wrath as a result of their lack of authority. The detrimental conduct of those who are subjugated is largely due to their helplessness and incapacity. Horizontal hostility refers to how women target other women whose professional success gives the impression of prominence [23]. Freire’s work has been taken in the study as underpinning theory by highlighting the concept of oppression in the workplace, such as gender discrimination and lack of administrative support. This theory encouraged questioning power structures and liberating the oppressed. Freire’s approach suggests raising awareness and promoting dialogue can address hostility as a symptom of deeper structural problems. There are three dimensions of horizontal hostility including work-related hostility, person-related hostility, and physically intimidating hostility. A person-related hostility has been described as ‘being ridiculed or humiliated related to work’, therefore, we studied person-related hostility among nurses who are considered to have a low status or less privileged job, and many stigmas are attached to this profession in Pakistan. By applying Frierean principles, the conceptual framework developed in the study can contribute to empowering individuals and fostering social change within organizational contexts.

### 2.1 Relationship between gender discrimination, person-related hostility, and intention to leave

Discrimination refers to the unfair treatment of individuals or groups based on gender, which can impact hiring decisions and promotions in the workplace [24]. Discrimination can occur in women at various stages along their career paths. Studies [25, 26] revealed gender disparities in salaries, income, and earnings, causing female workers to exhibit negative behavior. Males in formal employment earn higher rewards, are more stable, have more career opportunities, have easier access to profitable jobs, and earn higher salaries [27]. Gender bias in the workplace can lead to deep insecurity in working women, potentially leading to quitting their jobs [28]. Insecurity in jobs leads to employees becoming suspicious, anxious, and angry, resulting in emotional inelasticity, demotivation, and turnover intentions.

Two terms, sticky floor, and the glass ceiling are important to understanding gender discrimination in the workplace. These two factors also become the reason that causes female workers to perform hostile behavior. “Sticky floor” refers to the condition in which males and females having the same credentials and capabilities are probably hired to the same ranks or scale, but the appointment of males is at the upper scale and females at the bottom" [29]. Secretaries, nurses, or waitresses, who perform pink-collar jobs also experience sticky floors. The “glass ceiling” are measures that limit women’s career advancement beyond a certain point. Glass ceilings often affect women, who are highly educated and privileged, working at the middle management level, compared to women who come across sticky floors [30]. [31] highlights gender-discriminatory patterns in organizations as hindering women’s full leadership potential and limiting their opportunities for promotion and growth.

As a result of gender discrimination, intention to leave the organization develops and person-related hostility plays a direct as well as indirect role in developing it. Due to the development of person-related hostility victims start victimizing others through described negative behaviors as calling colleagues demeaning names, using expressions, speech tone, or gestures to degrade or tease them, demeaning their apprehensions, propelling them, and shoving things. [32] defined person-related hostility as covert violent behavior where the attacker aims to remain unseen to avoid conflict, social disapproval, conviction, or revenge. This indirect aggression includes gossiping, backbiting, spreading rumors, stalking, lying, and causing harm [33–35]. Therefore the presence of gender discrimination in the workplace causes enhanced person-related hostility which in turn causes the employee to leave the organization.

Based on the literature discussed above, the following hypothesis was formulated:

**H1**: Gender Discrimination is positively related to person-related hostility.
**H1a**: Person-related hostility mediates the relationship between gender discrimination and the intention to leave.

### 2.2 Relationship between lack of administrative support, person-related hostility, and intention to leave

[36] highlighted conflicts in healthcare workplaces due to respect issues, increased stress, responsibility refusal, and problematic duty determination, causing staff embarrassment. Disparities in education, discrimination, leadership, work environments, essential spaces, and diverse professional teams lead to conflicts, stress, and dissatisfaction [37, 38]. Stereotypes related to female dominance, task modifications, intellectual differences, and professional development differences in doctors and nurses’ professions contribute to conflicts, limited resources, mismanagement, administrative issues, and mentality differences [39]. [40] identified some common behaviors due to the presence of person-related hostility including non-verbal insinuation, verbal disrespect, discouragement, concealing facts, discordance, sabotage, scapegoating, betrayal, disregard for confidentiality, and broken promises. Person-related hostility occurs because of suppressed feelings of anger and hatred in oppressed individuals [41]. They express resentment through harmful conduct, including jealousy, gossip, insults, and accusations.

Administrative injustice results in a toxic work environment in which poor personnel management practices are applied by focusing on profit instead of people orientation. Due to a lack of administrative support, employees become frustrated and dissatisfied. A toxic work environment provides employees with a poor psychosocial climate, which results in conflict and aggression. [42] explained that managers in a toxic work environment damage the work environment through their deplorable human resource management practices. They damage staff self-confidence through reduced cooperation and information-sharing, and resultantly damage employee retention. They become impulsive and disrespectful toward their employees, creating a hostile work atmosphere. These managers found brilliance in their aggression and control. Hierarchal abuse also provides a theoretical basis for the origin of horizontal hostility owing to the importance of status [43, 44]. As a result of lack of administrative support, intention to leave the organization develops and person-related hostility plays a direct as well as indirect role in developing it. Therefore the lack of administrative support for women in the workplace causes enhanced person-related hostility which in turn causes them to leave the organization.

Based on the literature discussed above, the following hypothesis was formulated:

**H2**: Lack of administrative support is positively related to person-related hostility.
**H2a**: Person-related hostility mediates the relationship between lack of administrative support and intention to leave.

### 2.3 Person-related hostility and intention to leave

Increased stress, diminished productivity, increased absenteeism, and turnover are insightful and enduring outcomes of person-related hostility [45–47]. These authors argued that the prevalence of such hostile behaviours in the hospital setting has particularly challenged nurse leaders, as it enhances nursing dissatisfaction and turnover. Nursing education can be a powerful strategy to address workplace hostility. Respectful behavior at all ranks, modeling professionals, and confirming a supportive workplace emphasizing participative leadership and consensus building can be other strategies for reducing hostility among nurses. Moreover, Managers and educators should learn critical skills to teach new nursing graduates coping skills to deal with horizontal hostility. [48] conducted a qualitative study and found that 85% of participants experienced horizontal hostility in a healthcare setting. [49] used a mixed-method approach to determine the direct and mediated impact of bullying behaviors on nurses’ turnover intentions through job-related stress. The results of structural equation modeling via variance-based analysis showed significant direct and indirect effects of personal bullying on nurses’ turnover intentions through job stress. It was found that nurses experienced high levels of job stress due to exposure to bullying behaviors. Hostile behaviors occur recurrently, and their severity increases over time [50]. Such persistent negative behaviors result in continuous distress within individuals, leaving them helpless and incapable of dealing. It was suggested that nurse shortages might be worse in public sector hospitals due to these hostile behaviors, as nursing professionals who experience this intimidating behavior have more turnover intentions. In healthcare organizations, doctors’ hostile behavior toward nurses is a severe issue that needs to be addressed. Therefore the presence of person-related hostility in the workplace causes employees to leave the organization.

Based on the literature discussed above, the following hypothesis was formulated:

**H3**: Person-related hostility is positively related to the intention to leave.

### 2.4 Study’s conceptual framework

**Fig 1:**
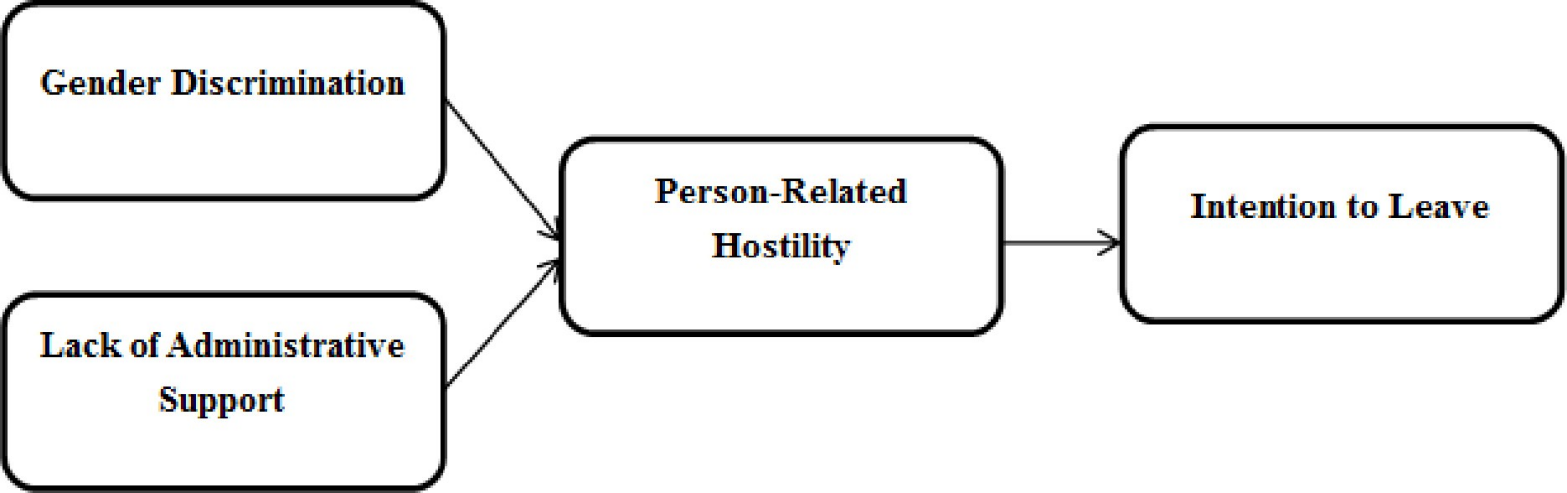
Conceptual framework.

From the literature discussed above, the conceptual framework (Fig 1) has been developed.

## 3. Methodology

### 3.1 Research design

A quantitative research design was applied, and questionnaire-based survey research was conducted. It is descriptive as it provides accurate and detailed information about the characteristics and behaviors of the target population. This is a cross-sectional questionnaire-based survey. For the target population, 14 hospitals from four main cities of Pakistan and a convenience sampling technique was used for data collection.

### 3.2 Participants and procedure

#### 3.2.1 Target population

This study analyzed the population of female nurses working in public sector hospitals in Pakistan. All these nurses have been registered by the Pakistan Nursing Council which regulates the affairs of the nursing sector in the country. In Pakistan, the nursing profession has mostly been overlooked by the health department resulting in a dire shortage of paramedics and nurses in the country. These nurses work under financial constraints and limited resources. The major issues they face include unmanageable workload, low pay, lack of career progression opportunities, low education and training opportunities, and hostile work environments. For the target population, 14 large public-sector hospitals, each with more than 300 female nursing professionals, were selected from Islamabad, Lahore, Faisalabad, and Peshawar. These hospitals provide healthcare services to many patients in all medical fields. In all these hospitals, 95% of nursing professionals were females who had been performing their duties as head, charge, and junior nurses. Medical Superintendents manage all administrative issues in hospitals, whereas chief nursing supervisors working as their subordinates manage the nursing workforce in all hospitals. Data were collected between August 2019 and January 2020.

#### 3.2.2 Study sample

The study surveyed female nurses in fourteen public-sector hospitals to measure person-related hostility, a dimension of horizontal hostility describing workplace harassment between females. Convenience sampling was applied and data were collected from female respondents who were easily accessible and willing to participate in the research. Due to the non-availability of the sampling frame, a non-probability sampling technique was applied. In this study, sample demographics were very similar to the population regarding age, designation, qualification, experience, clinical area and average hours worked showing a true representation of population characteristics. All the nurses worked in public sector hospitals with 200–300 beds.

#### 3.2.3 Procedure

For conducting this research study, formal permission was granted by the administration of all fourteen public sector hospitals. Medical Superintendents and Chief Nursing Superintendents of all hospitals formally allowed the researchers to collect the data. They forwarded requests to head nurses from different departments and asked them to facilitate the research. The researchers visited each hospital department and briefed the respondents about their research titles, objectives, and significance. Questionnaires were distributed to potential respondents after obtaining their face-to-face verbal consent. The data were collected in a natural setting. All public sector hospitals had a capacity of more than 300 beds. In public sector hospitals, nurses face complex situations due to limited resources and a large number of patients. Most registered nurses were willing to participate in the study and had more than one year of experience. The questionnaires were personally administered, and researchers visited all 14 hospitals themselves for data collection. Questionnaires were distributed to respondents who were willing to participate in the research survey. Social desirability bias was controlled through anonymity, framing questions appropriately, and total confidentiality. The researchers distributed questionnaires among respondents, allowed them to fill questionnaires as per convenience, and collected from them after one week. The researchers guaranteed the respondents about data confidentiality for honest answers. A total of 950 questionnaires were distributed to respondents in all hospitals. In total, 800 questionnaires were completed by respondents, with a response rate of 84%. For the final data analysis, 707 questionnaires were considered and included in the final analysis, of which 93 were incomplete and thus rejected. To avoid incomplete questionnaires, the respondents were briefed about the significance of this research for the nursing profession. The questionnaire was written in English. Validated scales were used for data collection, and responses were measured on a five-point Likert scale (strongly disagree, disagree, neutral, agree, strongly agree). Questionnaires that were partially filled out by respondents were rejected. As missing items may affect the reliability, validity, and analysis of the data. Because of the large sample size, no data handling method was applied, as rejection of questionnaires did not affect minimum sample requirements, data analysis, and generalisability of results. Some researchers have reported that sample sizes of 300 and 500 are suitable for factor analysis [51, 52]. G*Power software was used to confirm the sample size. The sample size was confirmed using G*Power software, with a 99% power and multiple correlations (R) of 0.40 at a significance level of 0.05.

#### 3.2.4 Data collection tool

To measure person-related hostility, we adapted the Negative Acts Questionnaire-Revised (NAQ-R) and added more items relevant to Pakistan. For cross-cultural adaptation, more items were included in NAQ-R related to the local context based on the qualitative research study. The questions were asked in the English language. [53] conducted a qualitative research study and explored behavioral tendencies of horizontal hostility as gossip, backbiting, negative comments, telling false stories, teasing and avoidance, verbal abuse, and non-verbal negative gestures among female nurses working in public sector hospitals of Pakistan. Based on this qualitative study, the NAQ-R was adapted, and twelve more questions regarding all these dimensions were added to measure person-related hostility in the context of Pakistan. For content validity, the instrument was discussed with subject area experts, and those items were included that represented all facets of person-related hostility. SPSS software was used for data coding and entry, and partial least squares equation modeling (Smart PLS-SEM) software was used to evaluate reliability, validity, and data analysis. To measure the reliability and validity of data, the values of Cronbach alpha, convergent validity, and discriminant validity were calculated. All these values were within acceptable range. The reliability and validity of the data were calculated using a reflective measurement model, whereas the hypotheses were tested using a structural equation model. For factor analysis, all items with outer loadings greater than 0.5 were included in the final analysis. Reliability was calculated using Cronbach’s alpha and composite reliability values, and validity was measured through convergent and discriminant validity. For convergent validity, all latent variables had an Average Variance Extracted of above 0.5. Participants’ attributes such as designation, qualification, marital status, and age were also considered in this study (Table 1). Junior nurses after 7 years of experience are promoted to the designation of charge nurses. Charge nurses work at the middle level of the hierarchy. Any other qualification includes MPhil and PhD in nursing.

**Table 1:** Demographics.

Characteristics	N (Percentage)
Designation	
Head Nurse	65 (9.2)
Charge Nurse	534 (75)
Junior Nurse	108 (15.3)
Marital Status	
Married	480 (67.9)
Single	227 (32.1)
Qualification	
Nursing Diploma (Matric)	31 (4.4)
Nursing Diploma (Intermediate)	114 (16.1)
Nursing Graduate (B.Sc.)	460 (65.1)
Nursing Graduate (M.Sc.)	38 (5.4)
Any Other	64 (9.1)
Age	
20–30 years	355 (50.2)
31–40 years	266 (37.6)
41–50 years	57 (8.1)
51–60 years	29 (4.1)
Institute	
PIMS	60 (8.5)
Poly Clinic Hospital	61 (8.6)
NIRM	16 (2.3)
SIMS	69 (9.8)
Lahore General Hospital	60 (8.5)
Jinnah Hospital	60 (8.5)
Sheikh Zayed Hospital	61 (8.6)
Mayo Hospital	61 (8.6)
Lady Willington Hospital	30 (4.2)
Allied Hospital	45 (6.4)
DHQ Hospital	54 (7.6)
Lady Reading Hospital	60 (8.5)
Peshawar General Hospital	30 (4.2)
Khyber Teaching Hospital	40 (5.7)

### 3.3 Measures

#### 3.3.1 Gender discrimination

Channar (2010) defined gender discrimination as discrimination or inequality towards a person or group because of identity or sex. Gender discrimination was measured using a six-item scale developed by Pejtersen et al. [54]. The items of gender discrimination were coded as GD1, GD2, GD3, GD4, GD5, and GD6.

#### 3.3.2 Lack of administrative support

A toxic work environment is caused by a lack of administrative support, where poor personnel management practices prioritize profits over people. Employees feel frustrated and dissatisfied because of contractual jobs, lack of authority, long working hours, and high job demands. A toxic work environment provides employees with a poor psychosocial climate that results in conflicts and aggression [55]. We measured the lack of administrative support using a 6 items scale developed by Pejtersen et al. [54]. Items related to lack of administrative support were coded as LAS1, LAS2, LAS3, LAS4, LAS5, and LAS6. [54] developed this instrument to measure the psychosocial work environment. Many researchers and work environment professionals used this instrument as it includes all facets of psychosocial work environment stressors and resources. We included items related to the dimensions of trust regarding management, justice, and social inclusiveness. The Cronbach alpha values for these dimensions were 0.80, 0.83, and 0.63 respectively.

#### 3.3.3 Person-related hostility

Person-related hostility has been measured as a dimension of horizontal hostility. [56] identified behaviors related to hostility such as gossiping, insulting, and yelling. We used a 19-item scale from the Negative Acts Questionnaire-Revised developed by [57]. This questionnaire contained items regarding gossip, backbiting, negative comments, telling false stories, teasing and avoiding, verbal abuse, and non-verbal negative gestures. The items of person-related hostility are codded as G1, G2, G3, BB1, BB2, NC1, NC2, TFS1, TFS2, TA1, TA2, TA3, TA4, TA5, VA1, VA2, NV1, NV2 and NV3. Einarson et al. (2010) developed the Negative Acts Questionnaire to measure workplace bullying having three dimensions such as work-related bullying, person-related bullying, and physically intimidating bullying. This instrument has also been used by researchers to measure horizontal hostility. The value of Cronbach’s alpha for person-related bullying was 0.90. In this study, twelve more items were added to measure person-related hostility in the context of Pakistan.

#### 3.3.4 Intention to leave

In the healthcare sector, intention to leave is a consequence of horizontal hostility. [58] described that maintaining a stable workforce is a vital resource that supports a competitive advantage for the success of organizations. They also found that retaining existing employees is cheaper than recruiting a replacement. We measured the intention to leave using the 3-item scale developed by Hinshaw et al. [59]. The items were coded as IL1, IL2, and IL3, respectively. [59] developed an instrument to measure turnover intentions among nursing staff to formulate retention strategies. The value of Cronbach’s alpha was 0.84. We used this instrument as it was developed with a focus on nursing administration to create innovative retention strategies to overcome the issue of turnover of staff and shortage of nurses.

### 3.4 Research ethics statement

Our study is part of the Ph.D. dissertation of one researcher and it was approved by the Advanced Studies & Research Board of the respective Institution (Letter of approval attached). It was related to the workplace issues of female nursing staff and did not include data collection from patients for medical research. This study was conducted with formal permission from all hospitals and nursing administration. The researchers informed respondents about the study’s aims, importance, confidentiality of the provided responses, and their ability to withdraw participation at any time if they felt uncomfortable. The questionnaires were distributed after obtaining verbal consent. This study reports the respondents’ perceptions, and there is no direct involvement of human objects in the experimentation.

## 4. Results

### 4.1 Descriptive statistics

Table 1 shows that most of the respondent nurses who participated in the study were charge nurses, comprising 75% of the sample, whereas 15% were junior nurses, and 9% were head nurses. Most respondents were married nurses (68%) and single nurses were 32%. Almost 65% of nurses had completed their BSc nursing, and 50% were young, between the ages of 21–30. Overall, there was a good mix of samples to depict a clear picture of the population.

### 4.2 Correlations

To measure concurrent validity, correlation coefficients were measured for all study variables. It measures the strength of the relationship between the two variables. We measured the Pearson correlation coefficient values for all variables (Table 2). The findings from the correlation support the study’s hypotheses that lack of administrative support (LAS) and gender discrimination (GD) have a positive impact on person-related hostility (PRH). Furthermore, the hypothesis that person-related hostility significantly impacts the intention to leave (IL) is supported.

**Table 2:** Pearson correlation coefficient.

Variables	1	2	3	4
GD	1			
LAS	0.667	1		
PRH	0.581	0.497	1	
IL	0.425	0.404	0.667	1

Lack of Administrative Support (LAS), Gender Discrimination (GD), Person-related Hostility (PRH), Intention to Leave (IL)

### 4.3 Reliability and validity

A reflective measurement model was used to evaluate data reliability and validity. We measured the reliability using Cronbach’s alpha and Composite Reliability. Convergent validity was measured using the Average Variance Extracted (Table 4).

#### 4.3.1 Outer loadings

Table 3 also displays the outer loading of each item. The outer loadings of all the items ranged from to 0.485–0.880. The outer loading of only one item was less than 0.5. All items’ outer loadings were higher than 0.5, except for Item 11 (LAS 5), whose outer loading was 0.485. This item is related to a lack of administrative support. We have deleted this item from the final analysis. Item 16 (BB1), which was related to person-related hostility, showed the highest outer loading of 0.825. The outer loadings of items 15, 16, 21, 24, and 29 were also above 0.80. Item 3 (GD3), which was related to gender discrimination, had the highest outer loading of 0.855. Items 4 (GD4) and 6 (GD6) had outer loadings greater than 0.80. Item 34 (IL3), related to the intention to leave, had an outer loading of 0.880.

**Table 3:** Outer loadings.

No	Item Code	Outer Loading	No	Item Code	Outer Loading
1	GD1	0.734	18	NC1	0.755
2	GD2	0.795	19	NC2	0.825
3	GD3	0.855	20	TFS1	0.792
4	GD4	0.843	21	TFS2	0.823
5	GD5	0.784	22	TA1	0.768
6	GD6	0.825	23	TA2	0.528
7	LAS1	0.762	24	TA3	0.800
8	LAS2	0.774	25	TA4	0.681
9	LAS3	0.768	26	TA5	0.511
10	LAS4	0.748	27	VA1	0.623
11	LAS5	0.485	28	VA2	0.793
12	LAS6	0.776	29	NV1	0.805
13	G1	0.679	30	NV2	0.773
14	G2	0.743	31	NV3	0.760
15	G3	0.800	32	IL1	0.869
16	BB1	0.802	33	IL2	0.871
17	BB2	0.785	34	IL3	0.880

Lack of Administrative Support (LAS), Gender Discrimination (GD), Person-related Hostility (PRH), Intention to Leave (IL)

#### 4.3.2 Internal consistency

To assess the instrument’s reliability, we measured its internal consistency using Cronbach’s alpha and Composite Reliability (Table 4). Cronbach’s alpha values ranged between 0.817–0.954 for all the latent variables. According to [60], a Cronbach’s alpha value greater than 0.70 shows high internal consistency. The composite reliability (CR) ranged between 0.868 and 0.959 for all the latest variables whereas recommended value of composite reliability is above 0.70.

**Table 4:** Construct reliability and validity.

Construct	Cronbach Alpha	Composite Reliability (CR)	Average Variance Extracted (AVE)	Discriminant Validity (Fornell- Larcker Criterion)			
				GD	LAS	PRH	IL
GD	0.893	0.918	0.651	**0.807**			
LAS	0.817	0.868	0.528	0.667	**0.727**		
PRH	0.954	0.959	0.555	0.581	0.497	**0.745**	
IL	0.845	0.906	0.762	0.425	0.404	0.667	**0.873**

Lack of Administrative Support (LAS), Gender Discrimination (GD), Person-related Hostility (PRH), Intention to Leave (IL)

#### 4.3.3 Convergent validity

Convergent validity measures construct validity. This shows that two or more items/constructs share variances in common that are assumed to be theoretically related. The average variance extracted (AVE) was calculated to measure the convergent validity (Table 4). All latent variables had AVE values higher than 0.5, ranging between 0.528–0.762. [60] recommended an AVE above 0.5 as the acceptance criteria. The findings showed that convergent validity was high for all latent variables.

#### 4.3.4 Discriminant validity

The differences between all latent variables were determined using discriminant validity. In any construct, the uniqueness of the manifest variable, in contrast to other variables in the model, was measured through discriminant validity. We applied the Fornell- Larcker criterion to measure discriminant validity. We measured discriminant validity by comparing the square root of average variance extracted (AVE) values with the correlation coefficient values among latent variables. The square root of the AVE values was greater than the correlation coefficients among the latent variables (Table 4).

### 4.4 Structural model and path coefficients

Direct relationships were measured in the first stage and mediation analysis was performed at the second level. In direct relationships, the impact of LAS and GD on PRH and the impact of PRH on IL were measured. In the mediation analysis, the mediating role of PRH in the relationship between LAS and GD with IL was examined. All direct and indirect relationships were significant (p < 0.05).

#### 4.4.1 Evaluation of collinearity

Collinearity was checked before testing the hypotheses. The structural model evaluates the significant level of collinearity between the explanatory and predictor variables. For all items, the variance inflation factor (VIF) was calculated to separately check the collinearity of each item. The VIF values were less than 5, indicating no collinearity issue, as recommended by Hair et al. (2017).

#### 4.4.2 Path coefficients of structural model

**Fig 2:**
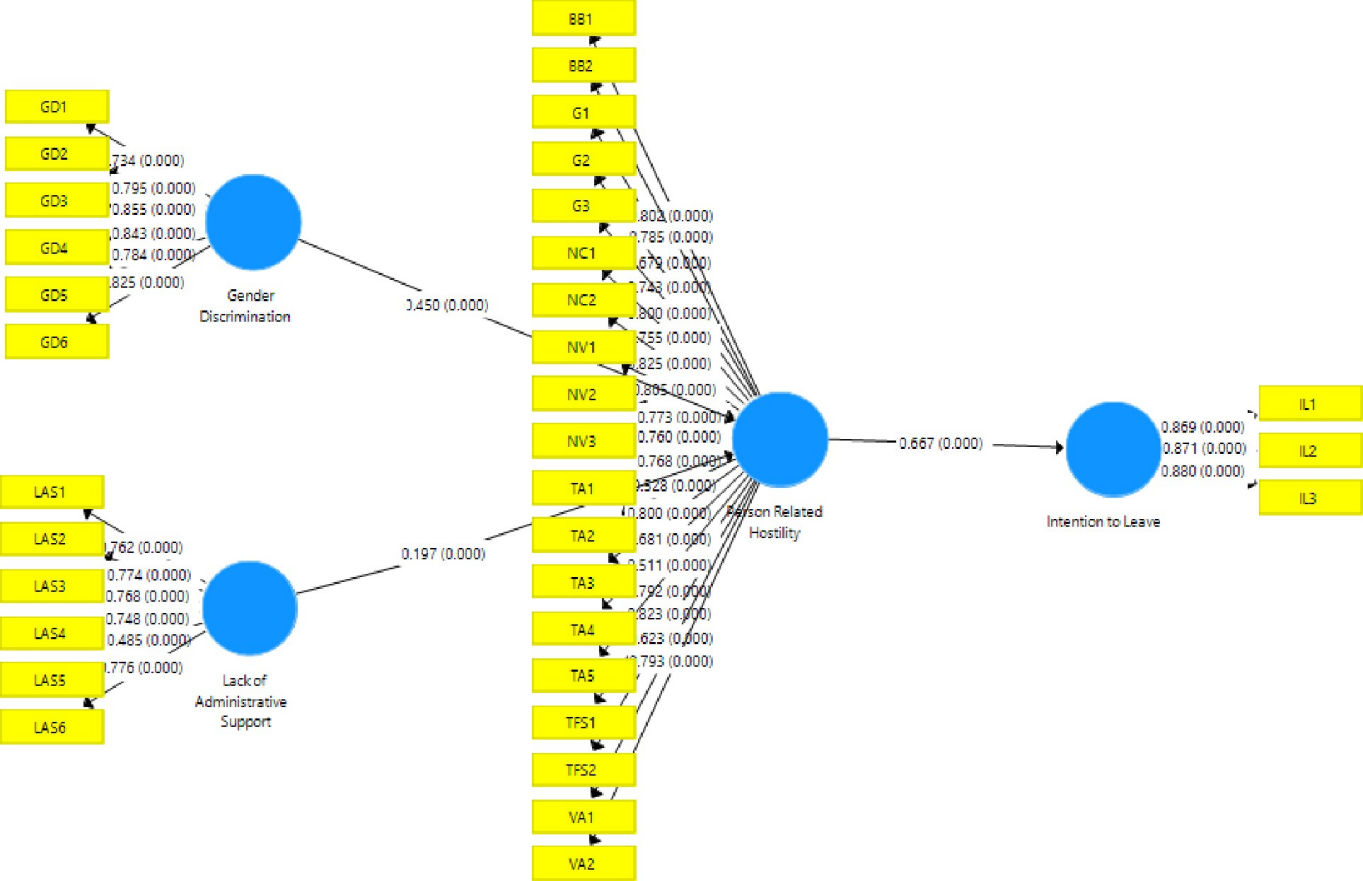
Path coefficients.

The significance of the association between independent and dependent variables was established by testing the hypotheses. β values were determined for each potential relationship in the proposed model. Larger values of β indicate a greater effect of the independent variables on the dependent variables. Table 5 displays the test results using the T-statistics and β values to corroborate the significance levels found throughout the analysis. The study found a significant positive association between gender discrimination and person-related hostility through direct path analyses (β = 0.450, p< 0.000), lack of administrative support and person-related hostility (β = 0.197, p< 0.000), and person-related hostility and the intention to leave the organization (β = 0.667, p< 0.000). In terms of mediating effects, we observed that person-related hostility mediated the relationship between gender discrimination and intention to leave (β = 0.300, p< 0.000) and lack of administrative support and intention to leave (β = 0.132, p< 0.000). Fig 2 shows the path coefficients.

**Table 5:** Path coefficients.

Structural Path	T-Statistic	Β	P-Value
GD -> PRH	9.944	0.450	0.000
LAS -> PRH	3.904	0.197	0.000
PRH -> IL	25.337	0.667	0.000
GD -> PRH -> IL	9.653	0.300	0.000
LAS -> PRH -> IL	3.722	0.132	0.000

Lack of Administrative Support (LAS), Gender Discrimination (GD), Person-related Hostility (PRH), Intention to Leave (IL)

#### 4.4.3 R^2^ and f^2^ effect size

The coefficient of determination (R^2^) was used to measure the overall effect size and variance explained by the dependent variable. Therefore, the coefficient of determination R^2^ measures the predictive accuracy of a model. We calculated R^2^ values for the dependent variables. According to the criteria provided by [61], the R^2^ values for person-related hostility (0.359) and intention to leave (0.446) were considered moderate. The variability in the R^2^ value when a construct is excluded or included from the model is determined by the f^2^ effect size. The F^2^ effect size describes the significant impact of the independent variable on the dependent variable when it is excluded from the model. The values of the F^2^ effect size were calculated for all independent variables. The findings showed a strong impact of lack of administrative support (f^2^ = 0.803) and gender discrimination (f^2^ = 0.175) on dependent variables.

#### 4.4.4 Evaluation of predictive relevance Q^2^

The prediction error was assessed using Q^2^ values, with a Q^2^ value above zero indicating the model’s predictive relevance with specific variables. For person-related hostility and intention to leave, the Q^2^ values were measured as (Q^2^ = 0.178) and (Q^2^ = 0.212) respectively. These values indicate a high predictive relevance for the selected dependent variables owing to the independent variables.

## 5. Discussion

This study primarily focused on highlighting the lack of administrative support and gender discrimination in the nursing profession, which turns female employees towards hostile behavior against other females in the workplace and causes organizational loss in the form of dissatisfied employees. The study, under the guidance of oppression theory, indicated that lack of administrative support and gender discrimination are positively linked to person-related hostility among nurses. Similarly, Person-related hostility significantly influences nurses’ intentions to leave their organization in the nursing profession. Moreover, person-related hostility mediates the relationship between gender discrimination, intention to leave, lack of administrative support, and intention to leave. The overall model of this study was tested in the context of developing nations. The study model can also serve as a guiding principle for managers of developed nations to retain female employees, but organizations must provide them with administrative support and a gender discrimination-free environment. If they fail to do so, the results can be disastrous because hostile employees will involve themselves in direct or indirect ruthless activities of bantering other female employees.

Reliability and validity analyses, internal consistency, convergent validity, and discriminant validity were calculated with values within the acceptable range, and the results supported the model. Results confirmed the reliability and validity of all instruments including the adapted NAQ-R used to measure person-related hostility among nurses in the context of Pakistan. The objective of this study was to highlight the organizational cultural factors such as gender discrimination and lack of administrative support that result in hostile behaviours among female nurses.

*Hypothesis H1 and H1a*

H1-H1a, both hypotheses were supported by the data analysis results and accepted in this study revealing a significant positive relationship between gender discrimination, person-related hostility, and intention to leave. These results are consistent with the findings of [14] where discriminatory practices were found as the main antecedent of person-related hostility among female nurses in public sector healthcare settings in Pakistan. Moreover, gender bias, disrespectful behavior of male staff, and delayed promotions negatively affected the psychological health of female nurses and resulted in anger, frustration, and hostile behaviors in healthcare settings [21]. Moreover, [62] described two types of stereotypes associated with nursing professionals’ gender and the nursing profession itself. They found nursing profession as female concentrated with low social status, skills, salary, autonomy, academic training, and entry-level requirements. This profession is viewed as subordinate to the medical profession. Findings from a qualitative study highlighted gender discrimination in nursing where respondents believed that career progression for males was favored over females. In healthcare settings, female nurses reported feeling invisible and were denied career progression opportunities offered to male nurses who were offered increased opportunities to attend seminars, workshops, and conferences [63]. This is the first study to provide empirical evidence of the indirect relationship between gender discrimination and person-related hostility and intention to leave. [15] argued that women’s self-esteem related to their competencies at the job might have been affected by well-documented hindrances and constraints called glass ceilings. As a result, they often internalize their lowered self-esteem, and the consequent adverse sensations and perceptions may force them to inflict horizontal hostility. Horizontal hostility occurs due to transgressing barriers, as it seems easy to confront colleagues horizontally instead of fighting vertically against oppressors. In the present study, we measured person-related hostility as a dimension of horizontal hostility that describes being ridiculed or humiliated regarding a person’s work. The study results also confirmed the earlier literature [25], where gender discrimination was a significant reason for creating workplace person-related hostility among nurses and building their intention to leave the organization. [64] found that nurses experience discrimination and gender-based hostility from other healthcare professionals at different ranks that adversely affect their health and retention. Lack of social and political power, weak position in an organizational hierarchy, and subordination by other professions were the main contributing factors to discrimination against nurses. Similarly gender discrimination and bullying are critical issues within healthcare organizations adversely affecting workplace environments, the well-being of nurses, and increased turnover.

*Hypothesis H2 and H2a*

Hypothesis H2-H2a was also supported in this study by the data analysis results. These findings are consistent with the literature [14] where a significant positive relationship was found between lack of administrative support and person-related hostility. Moreover, favoritism, nepotism, and lack of basic facilities were explored as contributing factors of horizontal hostility among female nurses [21]. Irrationally high workload, forced placement in different wards, work stressors, forced shifts, low salaries, poor working environment, denial of benefits for overwork, and lack of facilities result in hostility among nurses [65]. This is the first study to provide empirical evidence of the indirect relationship between lack of administrative support and person-related hostility and intention to leave. [66] described discrimination and lack of administrative support as the major clinical challenges faced by female nurses with grave consequences such as demoralization, fatigue, stress, loss of professional commitment, conflicts and hostility at work, and resignation. Female nurses experienced delayed promotions, gender discrimination, and cultural-organizational discrimination in the healthcare settings of Iran. Moreover, female nurses experienced discrimination, bullying, and harassment that adversely affected their psychological health and quality of life [67]. [68] described high levels of demand and effort, work overload, and low resources and rewards as the major factors of turnover intentions among nurses. Nurses experienced injustice or unfairness at work and hostility from colleagues, supervisors, and employers and consequently reported poor work climate and turnover intentions.

Our study is also in line with Price and Reichert [37], who described that in the healthcare workplace, conflicts that become the reasons for person-related hostility are work conditions, responsibility refusal, problematic determination of duties, and lack of support. Dissimilar education levels, discrimination and injustice, confrontational behavior toward leadership, complicated and unsatisfactory work environments, absence of essential spaces, and various cooperating teams of professionals also result in conflicts, stress, and dissatisfaction [38, 39]. Employees under horizontal hostility feel suppressed, angry, and hateful toward oppressed individuals; therefore, they express their resentment through harmful conduct, including jealousy, gossip, insults, and accusations.

*Hypothesis H3*

Hypothesis H3 was about the direct effect of person-related hostility on the intention to leave, which was also supported by the data analysis results. In our study, increased stress diminished productivity, and increased absenteeism in the form of turnover proved to be insightful and enduring outcomes of person-related hostility. It was also found that nurses experienced high levels of job stress because of exposure to gender discrimination and lack of administrative support which turned them into hostile behaviour. These behaviors occur recurrently, and their severity increases over time, resulting in continuous distress within individuals, leaving them helpless and incapable of dealing. Therefore, this behavior can create a shortage of nurses in public sector hospitals, because nurses who experience intimidating behavior have more turnover intentions [69]. Moreover, a significant positive relationship was found between horizontal hostility and turnover intentions among female nursing staff working in the healthcare sector of the Punjab region of Pakistan [70]. Our study is also in line with [71] who described turnover intentions as the adverse consequence of horizontal hostility and the main obstacle in nursing career development. In another study, [72] found a positive correlation between turnover intentions and person-related bullying among clinical nurses.

### 5.1 Implications for nursing management

This study has provoked the hospital administration and nurse managers’ role in reducing female nurses’ frustration and prohibiting hostility in the healthcare setting. Gender equality and supportive administration increase job satisfaction and the quality of patient care by preventing hostility and turnover intentions among female nursing professionals. It may help hospital administrators and nurse managers globally learn about the current situation of person-related hostility among female nurses in public sector hospitals in Pakistan. This may help them better understand cultural differences in horizontal hostility. Person-related hostility can be alleviated through equality, fairness, and the support of nurse managers and administrators. Nurse managers play a critical role in developing an amicable culture of group behavior and social support. This study may help to devise health sector policies to develop a supportive and collaborative workplace for female nurses. Moreover, this study has highlighted the importance that policymakers can enhance nurses’ job structure by providing better pay, training, and promotions, as well as study leaves for higher qualifications and timely health department promotions.

## 6. Conclusion and recommendations

The study investigates the prevalence of person-related hostility among female nurses and its relation with gender discrimination, lack of administrative support, and intention to leave. The study found that gender discrimination and lack of administrative support were positively associated with person-related hostility. The study also found that person-related hostility positively and significantly mediates the relationship between lack of administrative support, gender discrimination, and intention to leave. This study provides empirical evidence to support the assumptions that discriminatory practices and injustice result in frustration and hostility among female nurses. This hostile environment is the substantial reason that nurses develop their intention to leave the organization in the nursing profession. In this study, the NAQ-R was adapted and items regarding new dimensions of person-related hostility were added. All items were validated. HR strategies based on fair and equitable performance management and reward systems may be established to motivate competent female nurses. They should be provided with study leaves for higher education and training in developmental opportunities. Promotion criteria based on merit, timescale, and higher qualifications will reduce discrimination.

## 7. Limitations of the study

The present study has some limitations. Data was collected from main public sector hospitals located in the Federal Capital and two provinces of Pakistan including Punjab and Khyber Pakhtunkhwa. No response was received from the provinces of Sindh and Balochistan. The views of respondents from these two provinces might have impacted the findings of the study. In cross-sectional studies, causal relationships between variables cannot be determined. Therefore, to determine causal relationships, further study is required.

## Supporting information

S1 Data (RAR)
